# Integrating National Oral Health Programme and National Tobacco Control Programme in India: a concept for policy coherence

**DOI:** 10.3389/froh.2025.1574057

**Published:** 2025-04-11

**Authors:** Mithun Pai, Shweta Yellapurkar, Swapna Sarit, Kalyana C. Pentapati, Badekkila Ramachandra Avinash, Ramya Shenoy

**Affiliations:** ^1^ Department of Public Health Dentistry, Manipal College of Dental Sciences, Mangalore, Manipal Academy of Higher Education, Manipal, Karnataka, India; ^2^ Department of Oral Pathology and Microbiology, Manipal College of Dental Sciences, Mangalore, Manipal Academy of Higher Education, Manipal, Karnataka, India; ^3^ Centre for Cancer Epidemiology (CCE), Tata Memorial Centre (TMC), Navi Mumbai, Maharashtra, India; ^4^ Department of Public Health Dentistry, Manipal College of Dental Sciences, Manipal, Manipal Academy of Higher Education, Manipal, Karnataka, India

**Keywords:** oral health, policy coherence, policy integration, tobacco, tobacco program

## Abstract

**Introduction:**

Tobacco use continues to be a major risk factor for morbidity and mortality worldwide. At least 8.71 million fatalities were attributed to tobacco use, according to recent estimates from the Global Burden of Disease. Tobacco has a detrimental influence on oral health, such as oral cancer, periodontal disease, periimplantitis, and implant failure. This comparative analysis explores the potential integration of two programs—the National Oral Health Programme (NOHP) and the National Tobacco Control Programme (NTCP)—that are closely linked with each other.

**Possible blueprint for integrating NOHP and NTCP in India:**

joint awareness campaigns implementing a common risk factor approach, integrated screening and cessation services with dental colleges as tobacco cessation hubs, integrated oral cancer and tobacco screening as part of routine dental screening, and combination of monitoring and surveillance of tobacco usage and oral health.

**Challenges in integrating the NOHP and NTCP:**

Both the NOHP and NTCP face resource constraints relating to funding, human personnel, and infrastructure. These two initiatives are administered by separate branches within the health sector, involving different stakeholders with conflicting interests. There is a lack of unified data systems that provide a ground for comparing the effects of tobacco on oral health and integration of this data. The stigma associated with tobacco use: Tobacco users may be reluctant to associate themselves with oral health programs.

**Conclusion:**

Integrating the National Oral Health Programme and National Tobacco Control Programme in India can address the dual burden of tobacco use and oral health, by leveraging the strengths of both programs, which include educating trainers, raising awareness about oral health and tobacco use, improving access to trained manpower particularly dentists with dual roles, and promoting policy changes.

## Introduction

Tobacco use continues to be a major risk factor for morbidity and mortality worldwide. At least 8.71 million fatalities were attributed to tobacco use, according to the recent estimates from the Global Burden of Disease. Southeast Asia accounts for 81% of smokeless tobacco (SLT) users and 22% of smoked tobacco users aged 15 years and above (1). In the modern era, tobacco is the only legally permitted product that kills the majority of its users when consumed in any form. As per the World Health Organization, mortality due to tobacco-related diseases is higher than the combined mortality of the top three communicable diseases (tuberculosis, HIV/AIDS, and malaria) and is expected to rise considerably by 2030 (2).

In 2020, tobacco-related malignancies accounted for 27% of all cancer cases in India. By 2030, tobacco is expected to be responsible for over 8 million deaths annually, or 10% of all deaths worldwide, according to the WHO. The overall pooled estimate of any type of commercial tobacco use (CTU) among the Indian population across all age groups was 35.2% (CI 25.27–45.92). A recent pan-Indian survey by Bharahi et al. found that 49% of participants were in the 45–60 age group and 45% were in the 60+ age group. Among the participants, 39.8% smoked tobacco, 51.3% used SLT, and 8.9% both used SLT and smoked tobacco (3, 4).

Tobacco has a detrimental influence on oral health, other than oral cancer, such as increasing the risk of periodontal disease and dental implant-related issues such as periimplantitis and implant failure. Tobacco use also leads to dental caries, alveolar osteitis, and halitosis. Although these tobacco-induced diseases may be less fatal than oral cancer, they still impose considerable costs and health effects on people and the community, thus increasing the healthcare burden of the nation (5).

To enhance national oral health programs in the routine screening and management of tobacco users, several policy recommendations have been proposed to incorporate brief tobacco interventions into oral health programs. These recommendations align with the WHO Oral Health Program’s tobacco control and cessation policies (6).

Hence, this comparative analysis explores the potential integration of two programs—the National Oral Health Programme (NOHP) and National Tobacco Control Programme (NTCP)—that are closely linked with each other.

### The National Oral Health Programme (key takeaways)

The Government of India launched the NOHP to provide comprehensive oral healthcare within existing healthcare facilities.

Under the National Health Mission (NHM) component, states and union territories (UTs) can apply for assistance to establish dental care units at the district hospital level and below to provide oral health services to everyone through the program implementation plan (PIP) procedure. States and UTs may propose the following elements for support (7, 8).

The core focus of the program is to enhance oral health education, improve existing dental health systems, and strengthen the capability to detect and control oral diseases at the community level.

### The National Tobacco Control Programme (key takeaways)

The main activities of the different tiers of the NTCP can be summarized as follows:

In India, although there is already a well-organized national health program, called the NTCP, that is actively addressing this public health issue in a timely manner, it can be even more beneficial to investigate how to address this issue with the assistance of other national programs (2).

The goal is to reduce tobacco use through health education and interventions that help in quitting tobacco and the implementation of tobacco control laws, for example, the Cigarettes and Other Tobacco Products Act (COTPA). Furthermore, the program promotes education on the harmful aspects of tobacco use including its impact on oral cancer and other diseases of the mouth.

### Why integrate the National Oral Health Programme (NOHP) and National Tobacco Control Program (NTCP)?

Integrating the NOHP in India with the NTCP provides an excellent platform to counter the dual public health menace of tobacco use and tobacco-induced oral health problems. In India, tobacco use is widely considered a public health challenge leading to various oral diseases including, but not limited to, oral cancers, periodontitis, and tooth loss. The integration of both programs will improve the effectiveness of tobacco control interventions and the oral health status of the general population.

India has one of the highest tobacco consumption rates globally, with millions of users across diverse demographics engaging in both smoked (cigarettes and bidis) and smokeless (gutkha and khaini) forms of tobacco. These practices are a major cause of oral health problems particularly oral cancers and periodontal diseases accounting for high morbidity and mortality rates in the country. Integrating the NOHP and NTCP in India will help deal with the effect of tobacco on oral health and ensure a more organized and effective way of addressing these diseases.

### Possible blueprint for integrating the NOHP and NTCP in India

#### Joint awareness campaigns

Common risk factor approach: Create tobacco control campaigns that target the overall health and oral health of the population. These campaigns can propagate that stopping the use of tobacco has multiple benefits as tobacco is a risk factor for not only cancers and lung diseases but also oral cancers, gum diseases, or tooth loss.

#### Mass media and social marketing

The use of communication that includes, but is not limited to, television, radio, and social networks can help sensitize the community about the adverse effects of tobacco on oral health. This approach is important because in India, a multicultural society, these media campaigns can be exercised in different regional languages and cultures. Programs such as the Voice of Tobacco Victims (VoTV) campaign can be successfully utilized for such campaigns.

#### Community outreach

The public health pillar initiates community-friendly strategies in both rural and urban areas, with the aim of cessation of tobacco use and education on good oral hygiene practices. Mobile outreach programs that provide free services such as tobacco cessation therapy and oral health services can help gain confidence in populations that are difficult to reach.

#### Integrated screening and cessation services

Dental colleges and dental clinics as tobacco cessation hubs: Since most tobacco users visit dental clinics, these clinics are potential facilitators of tobacco cessation activities. Even without the existing interventions, dentists and dental hygienists can effectively be trained on how to conduct brief tobacco cessation counseling and make necessary referrals on cessation services to patients. Tobacco cessation intervention can be effectively implemented in dental colleges and dental clinics because of its relevance to dentistry and the ability to reach populations vulnerable to tobacco-related diseases. Tobacco use is associated with several oral health problems such as periodontal disease, tooth loss, and oral cancers. Hence, it is important to address tobacco consumption prevention and cessation during dental care delivery.

The need to incorporate tobacco cessation in dental practice and training presents an important public health intervention. As trusted healthcare providers, dentists build strong connections with patients allowing them to effectively support tobacco cessation. In this way, the tobacco cessation programs in dental colleges and clinics help dental professionals alleviate the burden of tobacco-related diseases and promote a healthy society devoid of tobacco.

Integration of oral cancer and tobacco use screening into routine dental visits: Screening for oral cancers and other tobacco-related oral diseases should be introduced during regular dentist visits. It is recommended that tobacco use should be screened among healthy patients, with cessation counseling or cessation referrals made during the dental visit. The routine visits should also include screening for early signs of oral cancer through inspection of abnormal growths, white or red lesions, or ulcers in the mouth, along with the examination of lymph nodes of the head and neck region. Screening and early detection of oral cancer drastically increase the survival rate of individuals, and dental health professionals are better suited to detect these changes in the oral cavity.

#### Training of healthcare providers

Oral health professionals as a cornerstone of tobacco cessation activities: Dental professionals, mainly dentists, play a role in tobacco cessation as part of their curriculum. They are proficient at tobacco cessation counseling because of the mandatory presence of tobacco cessation centers (TCC) in dental institutes or schools. As part of their curriculum, the dental students during their graduation would have taken up cases in TCC; hence, they will be oriented and better suited for both counseling and screening. The dentists then can train dental hygienists and dental assistants in tobacco cessation techniques. This could include motivational interviewing, offering nicotine replacement therapies, and educating patients on the importance of quitting tobacco for oral health.

#### Primary care and public health workers

Training primary healthcare workers (PHWs) in rural and urban areas is conducted to screen for tobacco use during routine health visits and to provide basic tobacco cessation support. These workers can be important in providing education on both oral health and tobacco cessation. This can be done by dentists who will be posted in the community health center or in any of the tertiary centers as part of the NOHP. This would provide a greater opportunity for both the dentists and healthcare workers to work closely and monitor the cessation program rather than an external person taking a one-off lecture for the same.

#### Monitoring and surveillance

Establishing a unified data collection and monitoring system will follow not only the trends of tobacco use but also the health intricacies associated with it. This system will take a holistic approach that utilizes different types of information, permits constant surveillance, and leads to actionable ways. The integrated data will be a useful tool for analyzing the trends in tobacco use and might form the basis for developing an artificial intelligence-based intervention system.

### The benefits of integrating the NOHP and NTCP

Aligning the national oral health policy of India with its national tobacco control policy, the financial implications are enormous. The major portion of allocated finances in NTCP is toward education and training of staff and healthcare providers, and the same is true for NOHP where a certain amount is to be allotted from flexipools from health system strengthening under the National Rural Health Mission (NRHM) (also known as Mission Flexible Pool). This integration of flexipools of both programs and effectively managing a bigger pool will be a good plan for better effectiveness of programs. In the long run, this would allow for a more productive workforce and less costs incurred on healthcare. Furthermore, the emphasis on health promotion and the opportunity for fresh revenue from taxes and foreign aid may create a strong base for the country's growth. Eloquently, the interrelation of oral health and tobacco control in its entirety is beneficial for India, allowing the country to adopt a more efficient and economically friendly public health model. The integration of tobacco control and oral health initiatives may increase the likelihood of sustained tobacco reduction efforts and improved oral health, as both issues are addressed comprehensively at the policy and grassroots levels.

### Joint awareness campaign for oral health and tobacco cessation

Such a campaign will be synergistic and will amplify the effects of both and will deliver a powerful message to the population. Tobacco intervention campaigns that include oral health messages and vice versa tend to have a wider population coverage. Individuals especially adolescents and young adults who are more concerned about esthetics and oral health but are not concerned with tobacco use may receive information on tobacco use which will help them know about the risks of tobacco.

### Sustainability of both programs

One of the factors that contribute to tobacco use and poor oral health is the existence of a common risk model that includes a bad diet, limited access to care, and other health determinants, integrating oral health with tobacco control programs. To minimize these risks, both programs have a common purpose which is to achieve a long-term effect on health and well-being. Tobacco control initiatives seek to decrease the population rates of smoking, whereas oral health initiatives seek to control the occurrence of oral diseases and improve hygiene practices. Because of this commonality of purpose, the chances that such measures will enhance one another are high resulting in a strong and sustainable program.

### Interdependent and synergistic effect on policy

Joint campaigns can generate support for public policies that limit tobacco use and promote better oral health, such as smoke-free laws, increased tobacco taxes, and greater access to dental care. By demonstrating the connection between tobacco and oral health, these campaigns can help build a stronger case for policy changes.

## Discussion: challenges of integrating the NOHP and NTCP

Resource constraints and resource limitations: The NOHP and NTCP have resource constraints relating to funding, human personnel, and infrastructure. While integration offers many benefits, there are multiple challenges related to funding sources, the type of funding, and capacity building particularly in rural areas that deserve special care.

The government sectors and stakeholders: The two initiatives are administered by separate branches within the health sector, involving different stakeholders with conflicting interests. These endeavors are often undertaken independently, which may require significant coordination and planning to ensure the smooth integration of the two most important programs that will form the basis of multiple policies.

There is a lack of unified data systems that provide a ground for comparing the effects of tobacco use on oral health and integration of this data in developing an effective preventive regime for effective policy for the prevention of oral disease and tobacco use. This may be because data collection in NTCP focuses on monitoring patterns of tobacco practice, health impacts of tobacco not necessarily on the oral cavity, and policy control on tobacco products.

The tobacco taboo: the stigma associated with tobacco use. Tobacco users may be reluctant to associate themselves with oral health programs as they may have low self-esteem. Hence, they might not attend programs integrating oral health and tobacco usage (9).

## Conclusion

Integrating the National Oral Health Programme and National Tobacco Control Programme in India aims to address the dual burden of tobacco use and oral health, by leveraging both programs' strengths which include educating trainers, raising awareness about oral health and tobacco use, improving access to trained manpower particularly dentists with dual roles, and promoting policy changes. An important step can be taken to reduce tobacco use, oral health issues, and the effects of tobacco use on the oral cavity and health in general. Such a combination of programs will not only enhance oral health status but also decrease the overall tobacco-induced health burden in the population. The process of integration of two national programs of great magnitude is a desired but difficult proposition as they have distinct objectives and differing goals, but on critical evaluation, the population at risk and the majority of training strategies align with each other. Hence, an evaluation to align the programs in a manner that can have a synergistic effect can be investigated as tobacco is a common risk factor for multiple diseases and oral health can be a greater beneficiary in the process.
